# Vaginal Lactoferrin Administration Decreases Oxidative Stress in the Amniotic Fluid of Pregnant Women: An Open-Label Randomized Pilot Study

**DOI:** 10.3389/fmed.2020.00555

**Published:** 2020-09-08

**Authors:** Alessandro Trentini, Martina Maritati, Valentina Rosta, Carlo Cervellati, Maria Cristina Manfrinato, Stefania Hanau, Pantaleo Greco, Gloria Bonaccorsi, Tiziana Bellini, Carlo Contini

**Affiliations:** ^1^Section of Medical Biochemistry, Molecular Biology and Genetics, Department of Biomedical and Specialist Surgical Sciences, University of Ferrara, Ferrara, Italy; ^2^Section of Dermatology and Infectious Diseases, Department of Medical Sciences, University of Ferrara, Ferrara, Italy; ^3^Department of Morphology, Surgery and Experimental Medicine, University of Ferrara, Ferrara, Italy; ^4^Section of Orthopedics, Obstetrics and Gynecology and Anesthesia and Resuscitation, Department of Morphology, Surgery and Experimental Medicine, University of Ferrara, Ferrara, Italy

**Keywords:** lactoferrin, oxidative stress, inflammation, amniotic fluid, pregnancy complications

## Abstract

**Background:** Oxidative stress (OxS) has been linked to several pregnancy-related complications. Previous studies demonstrated that lactoferrin (LF) has the ability to modulate inflammation, OxS and the immune function. Therefore, we aimed to observe whether vaginal LF administration was able to decrease OxS in the amniotic fluid (AF) of pregnant women undergoing mid-trimester genetic amniocentesis.

**Methods:** In this open-label clinical study, 60 pregnant women were divided into three groups: CONTROLS (*n* = 20), not treated with LF; LACTO 4HRS (*n* = 20), treated with LF 4 h prior to amniocentesis; LACTO 12HRS (*n* = 20), treated with LF 12 h prior to amniocentesis. Thiobarbituric acid reactive substances (TBARS), total antioxidant status (TAS) and oxidative stress index (OSI) were measured in AF samples. In addition, the *in vitro* antioxidant activity of LF on a cell line was tested.

**Results:** LF decreased the concentration of TBARS in the AF, with LACTO 4HRS demonstrating the lowest value compared with CONTROLS (*P* < 0.0001). LACTO 4HRS had higher TAS and lower OSI than CONTROLS (*P* < 0.0001 for both). *In vitro*, LF was effective against the oxidative challenge regardless of the time of pretreatment.

**Conclusion:** In conclusion, LF decreased both *in vivo* and *in vitro* OxS. LF administration may represent an intriguing clinical solution as an adjuvant to treat complications of pregnancy related to inflammation and OxS.

**Trial Registration:** Clinicaltrials.gov, NCT02695563. Registered 01 March 2016—Retrospectively registered, https://clinicaltrials.gov/show/NCT02695563.

## Introduction

The orchestration of a correct and controlled pro/anti-inflammatory milieu throughout pregnancy, together with the timed production of reactive oxygen species (ROS) and antioxidants, are essential features for a successful delivery (1, 2). ROS may be considered as end by-products of oxygen metabolism, where the presence of unpaired electrons in oxygen-containing molecules make them highly reactive and prone to attack biological structures (3). Normally, they are produced at low levels during the aerobic metabolism and act as second messengers involved in a plethora of physiological functions (4). As such, a balanced production of ROS is essential for several processes related to reproduction, starting from oocyte maturation, fetal development to delivery stimulation (1, 5). Importantly, the initial formation of ROS is limited due to the low oxygen tension in tissues, albeit increases when placenta starts to develop (6). The increase in ROS concentration triggers the synthesis of enzymatic antioxidants (e.g., Superoxide Dismutase, Glutathione peroxidase, Catalase) with the purpose of protecting the developing fetus from the new pro-oxidant environment (1). Therefore, in these initial stages of pregnancy, ROS function as signaling molecules driving the correct development of both fetus and its accessory structures, along with adaptations to the new environment (6). However, when the production of ROS is uncontrolled and exceeds the antioxidant capacity of a system to counteract their deleterious effects, the disruption of redox cellular homeostasis occurs, leading to a condition known as Oxidative Stress (OxS) (1). Indeed, a wealth of evidence confirmed that OxS may be directly or indirectly involved in several pregnancy-related complications such as spontaneous abortion, recurrent miscarriage, preterm labor and preterm pre-labor rupture of membranes (PPROM) (7). In particular, increased OxS markers have been found in serum of women with early pregnancy loss, that may/may not be accompanied by a decrease in enzymatic antioxidant defenses (8–11). OxS markers have also been found increased in the amniotic fluid (AF) of women with PPROM (12, 13) and connected with membranes senescence (14), which may lead to an increased susceptibility to proteolytic rupture.

In previous works, we demonstrated that vaginal lactoferrin administration is able to modulate the expression of inflammatory markers and Matrix Metalloproteases (MMPs), strongly involved in preterm labor, PROM and PPROM (15–17). Lactoferrin (LF) is an 80 kDa glycoprotein normally present in mammals, with a primary role as a defense mechanism against microbial infections (18). However, over the last decades LF has been discovered to play a role also in iron homeostasis (19), systemic inflammation/immune modulation and antioxidant processes (20, 21). These additional roles may be of particular importance to pregnancy-related complications. Owing these premises, in this work we aimed to determine whether vaginal LF administration is able to decrease OxS measured in the amniotic fluid of pregnant women undergoing mid-trimester genetic amniocentesis.

## Materials and Methods

### Study Design and Amniotic Fluid (AF) Collection

Since there is a lack of data in literature about the effect size of lactoferrin treatment on oxidative stress markers in the amniotic fluid, the sample size was calculated on the basis of previous results regarding interleukin-6 (IL-6) levels in the amniotic fluid of patients treated with lactoferrin (15). Considering the mean of the three groups being 1084, 242, and 1315 pg/ml (for group CONTROLS, LF 4HRS and LF 12HRS, respectively), with a within group SD of 1,100, the calculated effect size f was 0.419. With an alpha error of 0.05 and a power of 0.8 with three groups and an ANOVA setup, the required total sample size was 60, 20 patients each group. The calculations were made with the freeware software GPower 3.1.

Accordingly, in this open-label clinical study we enrolled 60 pregnant women undergoing genetic amniocentesis at 16th gestational week at the Obstetric Unit of Ferrara University from March 2011 to March 2012 (see CONSORT Flow Diagram in Supplementary Material). The inclusion criteria were: singleton physiological pregnancy and maternal age as the only indication to fetal karyotyping. The exclusion criteria were: assumption of drugs interfering with the immune system, previous miscarriages, pregnancy at risk due to maternal or fetal disease (e.g., diabetes, hypertension, pre-eclampsia, endometriosis, syndrome of polycystic ovary, symptomatology for acute vaginal infections). A questionnaire was administered to the patients in order to check for any complications (vaginal bleeding, uterine contraction, rupture of the membranes) within 7 days following the procedure. Accordingly, none of the enrolled patients demonstrated side effects or complications related with the procedures described in this study.

Eligible patients were randomly assigned in a 1:1:1 ratio with a random number table to receive a vaginal compound containing 300 mg of LF (Difesan, Progine Farmaceutici, Firenze, Italy) to obtain 3 groups:

- CONTROL: 20 untreated patients;
- LACTO 4HRS: 20 patients treated 4 h before amniocentesis with the compound;
- LACTO 12HRS: 20 patients treated 12 h before amniocentesis with the compound.

Amniotic fluid samples (total amount of 20 ml) were obtained by transabdominal amniocentesis and the sample not required for clinical purposes (roughly 2 ml) was centrifuged at 3,000 g for 10 min at 4°C to remove particulate material. The supernatants were then aliquoted and stored at −80°C until assay. No abnormalities were revealed by genetic analysis.

The research was approved by the local ethical committee and all the women gave their informed consent for the inclusion in the study. This study was carried out according to the ethical principles of the Declaration of Helsinki. This trial was retrospectively registered on Clinicaltrials.gov on 01 March 2016, trial number NCT02695563 (https://clinicaltrials.gov/show/NCT02695563).

### Fluorescent Thiobarbituric Acid Reactive Substances (TBARS) Assay

The assay of TBARS was carried out according to a published method with some modifications (22). Briefly, 50 μl of AF diluted two times with distilled water were mixed with 400 μl of 0.1% v/v phosphoric acid (Cat. No. W290017, Sigma-Aldrich, Milan, Italy) and 100 μl of 0.6% w/v 2-thiobarbituric acid (TBA, Cat. No. T5500, Sigma-Aldrich, Milan, Italy) in a microcentrifuge tube. The samples were incubated for 1 h at 95°C and the reaction was stopped by incubating the tubes 10 min in ice-water bath and centrifuged 10 min at 10,000 g and 4°C. Then, 200 μl of supernatant were dispensed in duplicate in a flat-bottomed black microtiter plate (Cat. No. 237108, Nunc, ThermoScientific, Texas, USA) and the fluorescence was read with a microplate fluorimeter (Tecan Infinite M200, Tecan, Switzerland) at excitation wavelength 530 nm and emission wavelength 565 nm. The concentration of TBARS, in Malondialdehyde (MDA) equivalents, was determined by interpolation with a standard curve made by different concentrations of 1,1,3,3-tetramethoxypropane (Cat. No. 108383, Sigma-Aldrich, Milan, Italy) within the range 0.0625–1 μM. The detection limit of the assay was 3 pmol with an intra- and inter-assay coefficient of variation below 10%.

The values were finally normalized by the total protein content in AF determined with the method of Bradford.

### Determination of the Amniotic Fluid Total Antioxidant Status (TAS)

The determination of amniotic fluid TAS was carried out according to a previous method present in literature (23) with some modifications. Hereon, Reagent 1 consisted in 0.4 M sodium acetate buffer, pH 5.8. Reagent 2 was prepared by dissolving 10 mM 2,2′-azinobis (3-ethylbenzothiazoline-6-sulfonate (ABTS, Cat. No. A1888, Sigma-Aldrich, Milan, Italy) in 30 mM sodium acetate buffer, pH 3.6, containing 2 mM hydrogen peroxide (Cat. No. 216763, Sigma-Aldrich, Milan, Italy). After a pre-incubation of 1 h at room temperature in the dark, the reagent was further incubated for 1 week at 4°C in order to complete the formation of the ABTS radical.

The assay was started by dispensing 20 μl of sample in duplicate in the wells of a flat-bottomed 96 wells microtiter plate (Cat. No. 655101, Greiner Bio-One, Italy), followed by the addition of 180 μl of Reagent 1 and 20 μl of Reagent 2. The absorbance was recorded at 740 nm (Tecan Infinite M200, Tecan, Switzerland) with two time points: 1) after 5 s mixing (sample blank); 2) after 5 min of incubation at room temperature. For the calculation of TAS in Trolox equivalents, the blank corrected reading (5 min-sample blank) of samples were interpolated with the data obtained with the same reaction where different concentrations of Trolox (Cat. No. 238813, Sigma-Aldrich, Milan, Italy), prepared in absolute ethanol within the range 0.03–2 mM, replaced the samples. The assay demonstrated an intra- and inter-assay variability below 5%.

### Determination of *in vitro* Antioxidant Effect of Lactoferrin

For the determination of the *in vitro* antioxidant effect of lactoferrin we used the histiocytic U937 cell line, routinely cultivated in complete medium composed of RPMI1640, 100 U/ml Penicillin-Streptomycin, and 10% Fetal Bovine Serum (FBS), at 37°C and 5% CO_2_ with 95% humidity. The cells (4^*^10^6^ per condition) were incubated in petri dishes and treated with 50 μg/ml lactoferrin for 4 or 12 h, or not treated as control. At the end of the incubation, the cells were harvested and centrifuged 5 min at 400 g, washed twice with Phosphate Buffered Saline (PBS, 150 mM NaCl, 10 mM NaH_2_PO_4_, pH 7.4) and incubated for 30 min with 250 μM hydrogen peroxide in Hank's Balanced Salt Solution (HBSS, 137 mM NaCl, 5.4 mM KCl, 0.25 mM Na_2_HPO_4_, 0.44 mM KH_2_PO_4_, 1.3 mM CaCl_2_, 1 mM MgSO_4_, 10 mM HEPES, 5.5 mM Glucose, pH 7.4). The cells were then harvested, washed twice with PBS and finally suspended in 90 μl of PBS. The cell suspension was lysed by sonication (Heat System Sonicator XL-2020) and centrifuged for 10 min at 800 g to remove particulate material. The supernatant was then collected and assayed for TBARS with the previous procedure, and for total protein content with the method of Bradford. MDA equivalents were normalized by the total protein amount and are reported as percentage with respect to the not treated control.

### Statistical Analysis

The normal distribution of variables measured in AF was checked with the Kolmogorov-Smirnov test. Since variables were not normally distributed, the comparisons between groups were made by the non-parametric Kruskall-Wallis test followed by the Mann-Whitney *U-*test, with Bonferroni correction for multiple comparisons. The OSI (Oxidative Stress Index) was calculated as the ratio MDA equivalents/TAS. For these analyses, SPSS 21 (IBM, USA) was used. *In vitro* data were analyzed by ANOVA with Tukey *post-hoc* test by using Graph Pad Prism v5.

For all the analyses, a *P* < 0.05 was considered significant.

## Results

### Baseline Characteristics of Patients

The mean maternal age in the whole population was 37.5 ± 2.1 years (minimum 35, maximum 42), and it was not different between the three groups (CONTROLS: 37.8 ± 2.4 years; LACTO 4HRS: 37.7 ± 2.3 years; LACTO 12HRS: 37.1 ± 2.1 years; *P* = 0.5). None of the patients included in the study suffered from other co-morbidities (e.g., diabetes, hypertension, endometriosis, syndrome of polycystic ovary, symptomatology for acute vaginal infections) ore used any antibiotic therapy or drugs that interfere with the immune system. All the patients were of white Caucasian ethnicity. The AF microbial cultures were negative in all the tested samples.

### Amniotic Fluid Markers

**Figure 1:**
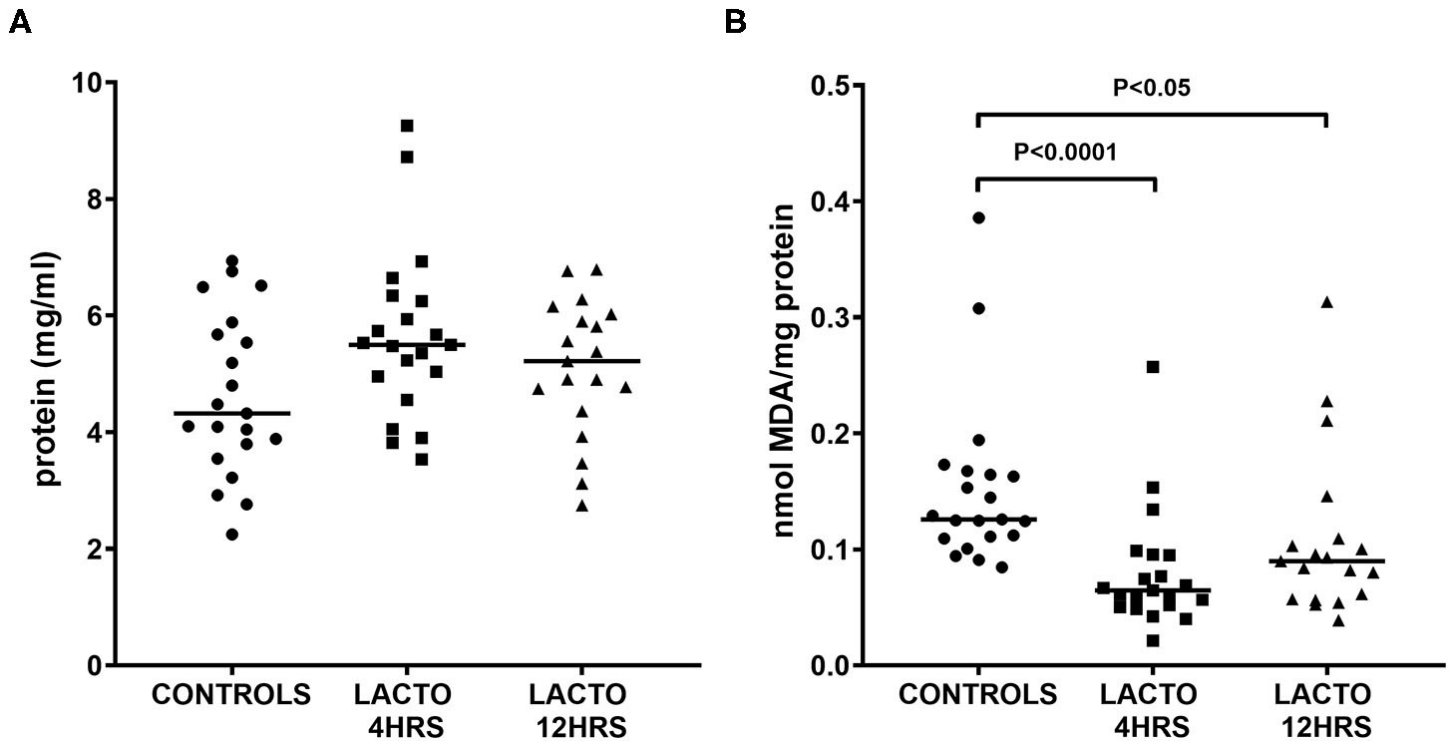
Protein concentration **(A)** and MDA equivalents normalized by total protein (TBARS, **B**), determined in AF of pregnant women undergoing amniocentesis. CONTROLS (*n* = 20): women not treated with lactoferrin; LACTO 4HRS (*n* = 20): women treated with lactoferrin 4 h before amniocentesis; LACTO 12HRS (*n* = 20): women treated with lactoferrin 12 h before amniocentesis.

As depicted in Figure 1, the concentration of total proteins was not different between the three groups (Figure 1A), but there was a significant decrease in TBARS concentration expressed as MDA equivalents (Figure 1B), for both LACTO 4HRS and LACTO 12HRS with respect to CONTROLS. In particular, the decrease in amniotic fluid MDA was more pronounced when patients were treated 4 h prior to the procedure (LACTO 4HRS vs. CONTROL, *P* < 0.0001), and it was still significant, though less pronounced, in women treated with lactoferrin 12 h prior to the procedure (LACTO 12HRS vs. CONTROL, *P* < 0.05). There was no significant difference between the two groups of women treated with LF (*P* = 0.416).

**Figure 2:**
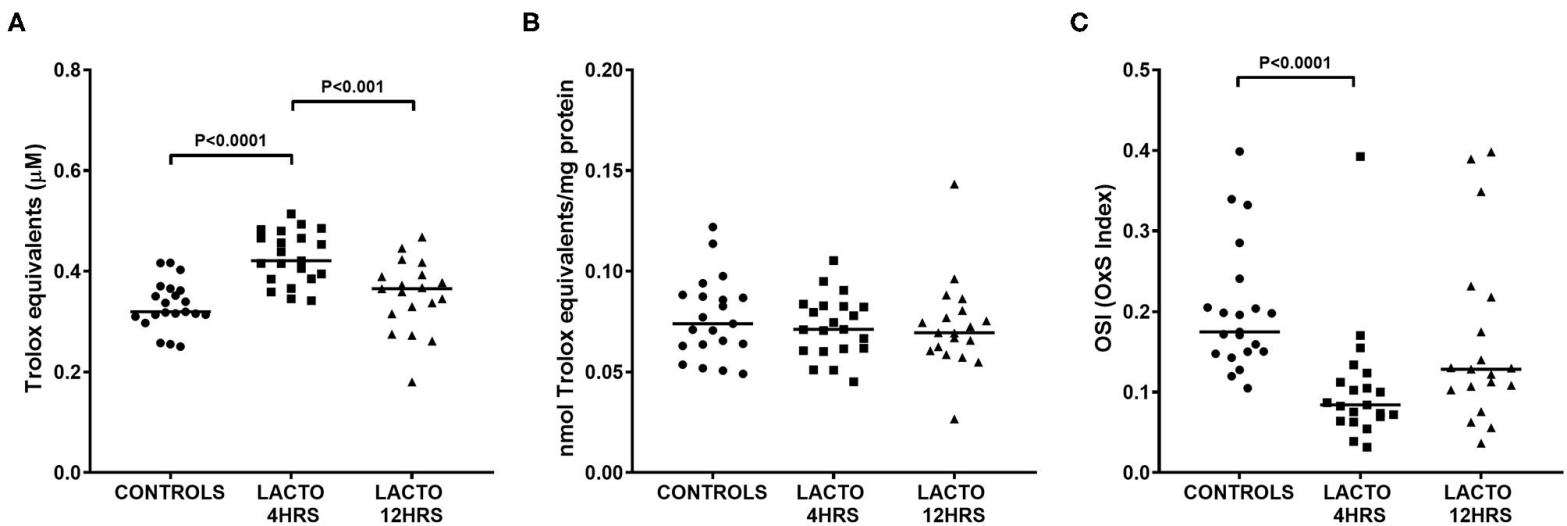
Total antioxidant status TAS **(A)**, TAS normalized by protein concentration **(B)**, and oxidative stress index (OSI, **C**) determined in AF of pregnant women undergoing amniocentesis. CONTROLS (*n* = 20): women not treated with lactoferrin; LACTO 4HRS (*n* = 20): women treated with lactoferrin 4 h before amniocentesis; LACTO 12HRS (*n* = 20): women treated with lactoferrin 12 h before amniocentesis.

The total antioxidant status was significantly increased only in women treated with lactoferrin 4 h prior to amniocentesis (Figure 2), with values almost 35% higher than CONTROLS (Figure 2A, *P* < 0.0001) and 17% higher than LACTO 12HRS (Figure 2A, *P* < 0.001). On the contrary, TAS in LACTO 12HRS and CONTROLS did not change. Of note, the differences we found disappeared after adjustment for total protein concentration in AF (Figure 2B). Nonetheless, the OSI (oxidative stress index), a measure of the oxidant status and of the balance between oxidants and antioxidants, was significantly lower only in the group LACTO 4HRS compared with CONTROLS (Figure 2C, *P* < 0.0001).

### *In vitro* Antioxidant Effect of Lactoferrin

**Figure 3:**
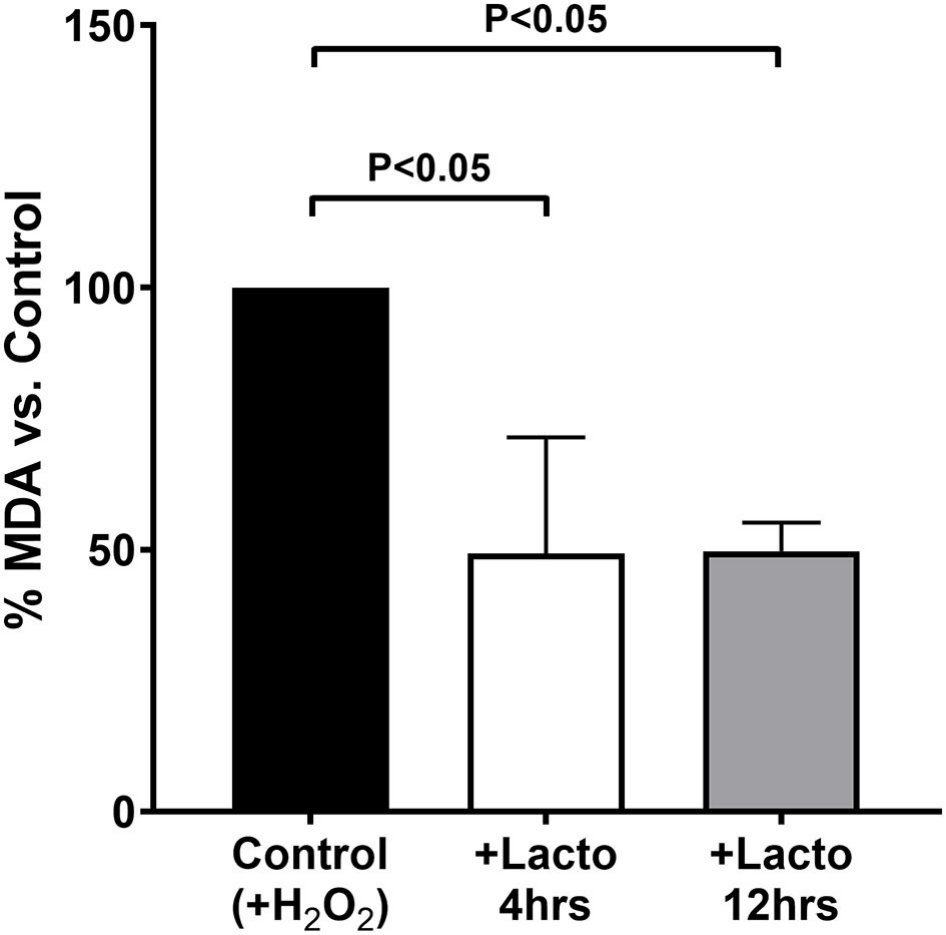
MDA equivalents measured in cells treated 4 h (open bar) or 12 h (gray bar) with lactoferrin, or not treated as a control (black bar), and subjected to oxidative challenge with hydrogen peroxide. Values are normalized by the control and the data represent the mean ± SD of 3 independent experiments.

The antioxidant effect of LF was tested *in vitro* by treating cells for two incubation times, 4 and 12 h, resembling those performed on pregnant women, to observe whether the protein lost its efficacy after prolonged incubation time. As reported in Figure 3, both short and long incubation times were effective in decreasing the production of TBARS when the cells were challenged with hydrogen peroxide (*P* < 0.05 for both treatments vs. Control). In addition, there was no substantial difference between 4 and 12 h of treatment with lactoferrin, confirming that the protein is effective independently from the incubation time.

## Discussion

The findings of the present study suggest that vaginal administration of LF is able to modulate OxS, in particular by interfering with the production of lipoperoxidation products. This may be due to a direct or indirect action of LF on ROS formation. The antioxidant properties of LF have been attributed mainly to its ability to sequestrate Fe^3+^ from the environment making it unavailable for the Fenton reaction, which promotes the formation of a variety of highly toxic ROS (1). Indeed, LF has high affinity for free iron (III) and this is crucial for its bacteriostatic activity against iron-dependent microorganisms (24). Moreover, LF also demonstrate a direct bactericidal activity against Gram-negative organisms due to high affinity for lipopolysaccharides (LPS) (25).

Further recent studies suggested that LF has also a direct ROS-scavenging ability independent from its chelating nature (26). Our *in vitro* results confirmed the previous results, by showing that LF is able to decrease lipoperoxidation products when cells are challenged with an oxidant such as H_2_O_2_. In addition, from our results we confirmed that LF efficacy is not diminished even after a prolonged incubation of cells with the protein. Therefore, the *in vivo* finding that women administered with LF 12 h before amniocentesis had levels of TAS and OSI comparable with controls, but still a significant MDA-lowering activity, may be due to the short *in vivo* half-life of the protein (15). This is in line with our previous observations indicating that LF seems more effective at 4 h rather than 12 h of administration (16, 17). Thus, from a mechanistic point of view, once LF enters the amniotic compartment it can decrease the formation of lipoperoxides by directly scavenging intermediary ROS, or by “removing” Fe^3+^ from the Fenton reaction (27, 28).

OxS is increasingly acknowledged as one of the key contributors, in mutual association with inflammation, of several pathologies ranging from, but not limited to, metabolic to degenerative diseases (3, 29, 30). The same holds true for pregnancy-related complications, where the redox imbalance is considered as an underlying factor of conditions like PPROM, pre-term labor or recurrent miscarriage (1, 7). The maternal immune system undergoes significant modulation during pregnancy, a task that is also performed by the fetus itself. In fact, pregnancy success seems to be ensured through a delicate balance of pro and anti-inflammatory cytokines, chemokines, prostanoids, and ROS, which in concert modulate the maternal response to fetal invasion through the production of MMPs (31) and prevent infection from a multitude of potential pathogens.

It has been documented that an increase in OxS is physiological within the 2nd trimester of pregnancy, which is supported by a mitochondrial production of ROS within the placenta (1). However, the striking link between OxS and pregnancy complications advocates against an uncontrolled formation of ROS, urging the need of therapies able to effectively control the detrimental effects of such toxic species. Indeed, up to now clinical trials employing antioxidants supplementation (mainly vitamin C and vitamin E) to reduce OxS and improve pregnancy outcomes have failed to detect a real benefit (32–34). Multiple reasons may be listed for this unexpected outcome (35), but it is more likely that the heterogenous nature of ROS together with the indissoluble link between inflammation, OxS, and OxS-related tissue damage can be the determinant factor. Thus, it is in our view that molecules able to counteract OxS and, at the same time, to modulate inflammation and the immune responses may be the key for a successful reduction of complications. Lactoferrin easily fulfill these criteria. In fact, our previous studies demonstrated that LF administration is able to modulate inflammatory markers in the amniotic fluid, including the detrimental MMPs connected to pre-labor rupture of membranes (PROM) and PPROM (16, 17). In addition, there is overwhelming evidence that LF can normalize insult-induced reactions due to immune modulation properties (21). However, whether a prolonged vaginal administration of LF may be able to decrease the frequency of such complications and pre-term labor as well has yet to be established. Therefore, further clinical studies are needed on this matter.

This study was not without limitations. First, the small sample size may have affected the reliability of our conclusions. Second, the cross-sectional nature of the study, as opposed to the longitudinal approach, precluded us to draw any causative relationship between LF treatment and possible long-term benefits against pregnancy-related complications. Third, the measurement of MDA through the evaluation of thiobarbituric acid reactive substances rather than measuring also 8-isoPGF2α (8-isoprostane) concentration, which is considered the gold standard for lipid peroxidation, may have affected our conclusions. However, it has been reported that both measures correlate well in plasma (36), which contains more interfering substances than AF.

In conclusion, our results demonstrated that transvaginal LF is able to directly control the *in vivo* and *in vitro* formation of ROS, which adds to the already known immunomodulatory actions of the protein. Thus, from our data, although with some limitations due to the cross-sectional nature of this study, we can hypothesize that LF administration may represent an intriguing clinical tool as an adjuvant to treat complications of pregnancy related to inflammation and OxS. However, further studies with a longitudinal setup and larger populations are mandatory to fully understand the real impact that a continuous regimen of LF administration may have on pregnancy complications.

## Data Availability Statement

The raw data supporting the conclusions of this article will be made available by the authors, without undue reservation.

## Ethics Statement

The studies involving human participants were reviewed and approved by Comitato etico della provincia di Ferrara. The patients/participants provided their written informed consent to participate in this study.

## Author Contributions

AT and CCo: conceptualization and project administration. VR: methodology. AT: software, formal analysis, data curation, writing—original draft preparation, and supervision. SH, CCe, PG, GB, TB, CCo, MCM, and MM: writing—review and editing. All authors have read and agreed to the published version of the manuscript.

## Conflict of Interest

The authors declare that the research was conducted in the absence of any commercial or financial relationships that could be construed as a potential conflict of interest.
