# Supplementary figures and images for: Vaginal Lactoferrin Administration Decreases Oxidative Stress in the Amniotic Fluid of Pregnant Women: An Open-Label Randomized Pilot Study
Source: Front Med (Lausanne). 2020 Sep 8;7:555. doi: 10.3389/fmed.2020.00555 (PMC7505926; doi:10.3389/fmed.2020.00555)

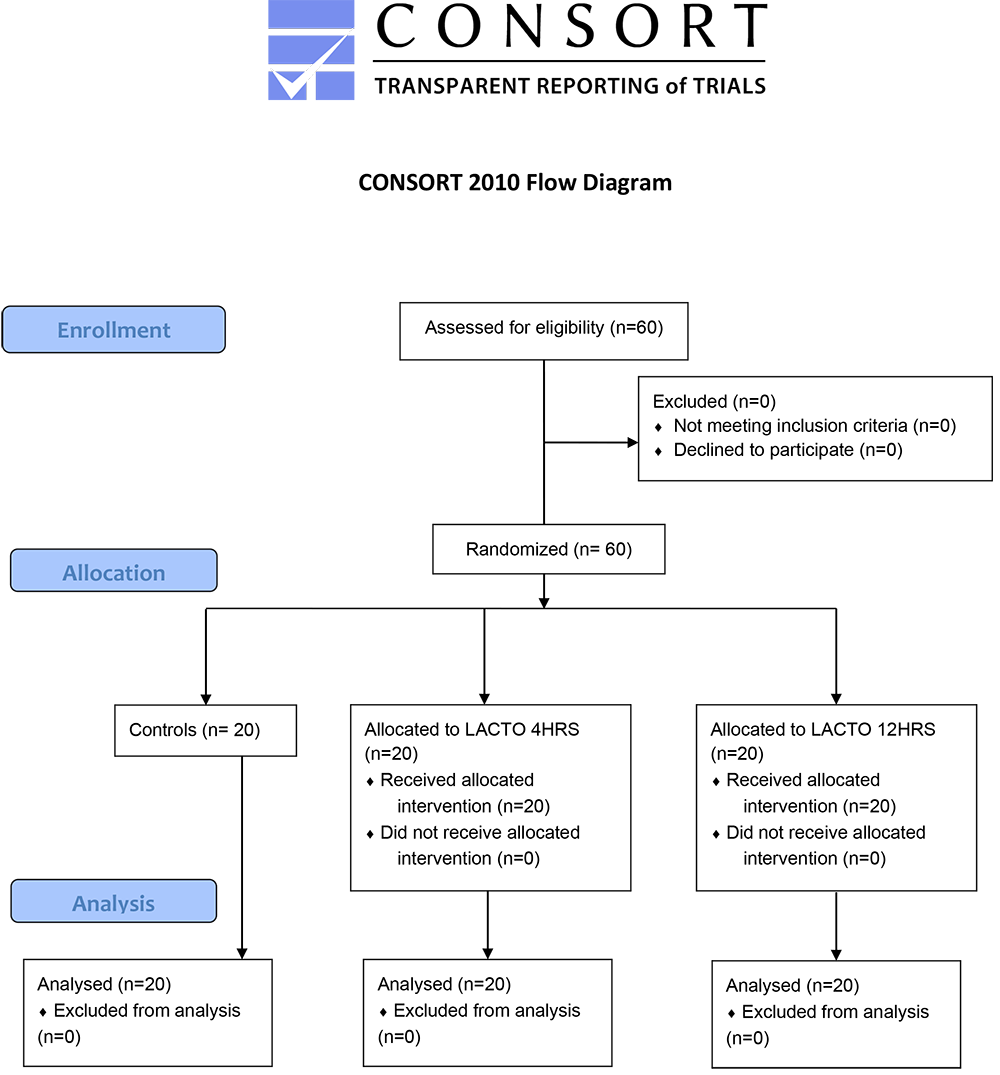

Supplement: Supplementary file 1 [file Image_1.TIF]
